# Psychological Treatment of Low Sexual Desire in Women: Protocol for a Randomized, Waitlist-Controlled Trial of Internet-Based Cognitive Behavioral and Mindfulness-Based Treatments

**DOI:** 10.2196/20326

**Published:** 2020-09-29

**Authors:** Milena Meyers, Jürgen Margraf, Julia Velten

**Affiliations:** 1 Clinical Psychology and Psychotherapy, Mental Health Research and Treatment Center Faculty of Psychology Ruhr University Bochum Bochum Germany

**Keywords:** sexual desire, sexual dysfunction, women’s sexual health, cognitive behavioral therapy

## Abstract

**Background:**

Psychological therapies are effective treatments for hypoactive sexual desire dysfunction (HSDD; formerly hypoactive sexual desire disorder), a common sexual dysfunction among women. Access to evidence-based treatments, however, remains difficult. Internet-based interventions are effective for a variety of psychological disorders and may be a promising means to close the treatment gap for HSDD.

**Objective:**

This article describes the treatment protocol and study design of a randomized controlled trial, aiming to study the efficacy of cognitive behavioral and mindfulness-based interventions delivered via the internet for women with HSDD to a waitlist control group. Outcomes are sexual desire (primary) and sexual distress (secondary). Additional variables (eg, depression, mindfulness, rumination) will be assessed as potential moderators or mediators of treatment success.

**Methods:**

A cognitive behavioral and a mindfulness-based self-help intervention for HSDD will be provided online. Overall, 266 women with HSDD will be recruited and assigned either to one of the intervention groups, or to a waitlist control group (2:2:1). Outcome data will be assessed at baseline, at 12 weeks, and at 6 and 12 months after randomization. Intention-to-treat and completer analyses will be conducted.

**Results:**

We expect improvements in sexual desire and sexuality-related distress in both intervention groups compared to the waitlist control. Recruitment has begun in January 2019 and is expected to be completed in August 2021. Results will be published in 2022.

**Conclusions:**

This study aims to contribute to the improvement and dissemination of psychological treatments for women with HSDD and to clarify whether cognitive behavioral and/or mindfulness-based treatments for HSDD are feasible and effective when delivered via the internet.

**Trial Registration:**

ClinicalTrials.gov NCT03780751; https://clinicaltrials.gov/ct2/show/NCT03780751

**International Registered Report Identifier (IRRID):**

DERR1-10.2196/20326

## Introduction

### Background

A sexual dysfunction can be described as a disturbance in a person’s ability to respond sexually or to experience sexual pleasure that leads to significant personal distress [1]. One of the most common sexual dysfunctions among women is a lack of sexual desire which, according to the eleventh edition of the International Classification of Diseases (ICD-11), can manifest as reduced or absent spontaneous desire (sexual thoughts or fantasies), reduced or absent responsive desire to erotic cues and stimulation, or an inability to sustain desire or interest in sexual activity once initiated [2]. If a pattern of low sexual desire is present over a period of at least several months and is associated with clinically significant distress, a hypoactive sexual desire dysfunction (HSDD; formerly hypoactive sexual desire disorder) can be diagnosed. A UK study found that among 6777 sexually active women, 34.2% experienced low desire [3]. When applying the morbidity criteria listed in the fifth edition of the Diagnostic and Statistical Manual of Mental Disorders (DSM-5), 6.5% of women indicated low sexual desire or arousal for several months within the last year, and 0.6.% met the criteria for female sexual interest/arousal disorder [4]. Low sexual desire does not only affect women’s sexual health but also has detrimental effects on their quality of life [5]. Individuals with sexual dysfunctions (including low desire) are more likely to report higher rates of sexually transmitted infections, unwanted sex, unemployment, relationship breakdown, and difficulties discussing sexuality [3]. In addition, low sexual desire is often comorbid to mental health problems such as depression and anxiety [6,7].

### Treatment of Low Sexual Desire

Low sexual desire has received widespread attention from clinicians, researchers, and the lay public because of its high prevalence and its seeming resistance to treatment [8] which led to increased efforts to find a “Pink Viagra” (ie, an effective pharmacological agent) [9]. To date, flibanserin is the only drug that has received approval for treatment of low sexual desire in women in the United States and Canada. While this drug, which has pharmacological effects on serotonin and dopamine receptors [10], was being tested on patients with major depressive disorder, its positive effect on women’s sexual functioning was discovered [11]. So far, 3 systematic reviews or meta-analyses have found modest positive effects on women’s low sexual desire [12-14], while also acknowledging its side effects (eg, nausea, dizziness, fatigue). There has also been renewed interest in the advancement of psychological treatments for women’s low sexual desire [9]. A meta-analysis found psychological interventions to be effective treatments for low sexual desire [15] and effects for symptom reduction were large compared to waitlist controls (*d*=0.91; 95% CI 0.38-1.45; *P*=.012). Furthermore, such treatments lead to medium to large increases in sexual satisfaction.

### Components of Psychological Treatments for Low Sexual Desire

Psychological interventions for low desire often comprise a variety of components such as psychological and sexual education, couples exercises, guided masturbation, communication training, cognitive behavioral as well as mindfulness-based techniques [15]. Many interventions aim to educate women about their genital anatomy, psychological and physiological aspects of sexual arousal, and sexual difficulties (eg, symptoms, prevalence). Other common treatment elements are self-exploration and sensate focus. Self-exploration may include exploration of genital anatomy using a handheld mirror. Self-touch exercises aim to provide knowledge about how to elicit pleasurable sexual feelings and arousal (Brotto, Paterson, Basson, Driscoll, and Grabovac, unpublished manual, 2015 and unpublished manuscript, 2015). Sensate focus comprises gradually progressing exercises with varying degrees of touch, administered to a partner’s body [16]. Exercises start with a couple taking turns at providing nonsexual touch and end with sexual intercourse.

Even supposedly specialized treatments often include a broad range of psychological methods [9]. Four modules of mindfulness-based treatment, for example, included not only mindfulness exercises but also information on sexual dysfunctions, sexual response, and female genital anatomy, supplemented by self-exploration, cognitive restructuring, and sensate focus exercises [17]. Thus, it remains unclear which of the specific treatment components are the main drivers of change and more research is needed to understand which methods and techniques are necessary and sufficient to improve women’s low sexual desire.

Among the most common treatments for low desire are cognitive behavioral therapy (CBT) and mindfulness-based therapy (MBT) [15]. CBT is change oriented and is one of the most researched forms of psychotherapy. It is effective for a variety of mental health problems such as depression, anxiety disorders, and posttraumatic stress disorder [18], as well as sexual dysfunctions such as HSDD [15]. MBT is an acceptance-based approach using mindfulness, an ancient Eastern practice with roots in Buddhist meditation, defined as present-moment, nonjudgmental awareness [19], and intends to shift the focus of attention to one’s body and breath. MBT has been found effective for the treatment of depression, anxiety, and stress [20]. Evidence for its effectiveness for women’s sexual dysfunctions stems from a few, mostly uncontrolled, studies [17,21,22].

### Internet-Based Interventions

Although many women are distressed by their lack of desire, the number of those who receive qualified, evidence-based treatment remains low [23]. Sexual dysfunctions are connected to stigma and reluctance in seeking professional help [24,25]. Furthermore, a lack of information on the treatment options prevails as well as structural barriers, such as limited access to qualified therapists. This issue could be addressed by developing and disseminating treatments that require less direct forms of contact, namely internet-based interventions. A growing body of literature suggests that interventions delivered via internet or mobile technology are feasible and effective to improve depression, anxiety [26], and even psychosis [27]. These interventions offer varying degrees of guidance ranging from self-help, user-led interventions, to regular to contact with a trained clinician. Interventions involving at least some kind of guidance or coaching have been proven to be more effective than unguided interventions [28]. Concerning internet-based interventions for sexual dysfunctions in women, van Lankveld [29] reviewed 5 studies and found that these interventions were effective in improving sexual functioning and emotional intimacy in couples. While 2 of these studies also included women with low sexual desire [29], treatments did not focus on improvement of sexual desire but targeted a broad range of women’s sexual difficulties (eg, anorgasmia, genital pain). Thus, no studies have evaluated the efficacy of internet-based CBT (I-CBT) or MBT (I-MBT) designed for the treatment low sexual desire in women.

### Study Aim

The aim of this study is to present the protocol of a randomized controlled trial (RCT) comparing 2 internet-based interventions for low sexual desire in women with HSDD to a waitlist. Hypotheses 1 and 2 are that at posttreatment (12 weeks after randomization), women receiving I-CBT or I-MBT report significantly more sexual desire (primary outcome; H1) and less sexuality-related distress (secondary outcome; H2) compared to a waitlist control condition. It is also proposed that these differences are maintained at the 6- and 12-month follow-up assessments. Differences in outcomes between I-CBT and I-MBT at the 12-week-, 6-month, and 12-month assessments are inspected on an exploratory basis. It is also explored whether improvements from pretreatment to posttreatment are dependent on treatment dosage (eg, completed modules, time spent with at-home exercises) and whether changes in proximal variables such as mindfulness or rumination mediate improvement in symptoms in either one of the treatments. Whether improvements in symptoms are predicted by changes in sexuality-related interpretations and associations assessed with computerized reaction-time paradigms (ie, Single-Target Implicit Association Test [STIAT] and Scrambled Sentences Task [SST]) is examined as well. To explore participants’ perceptions of the treatments more thoroughly, qualitative telephone interviews will be conducted with 25 participants in the I-CBT and I-MBT condition, respectively.

## Methods

### Design

**Figure 1:**
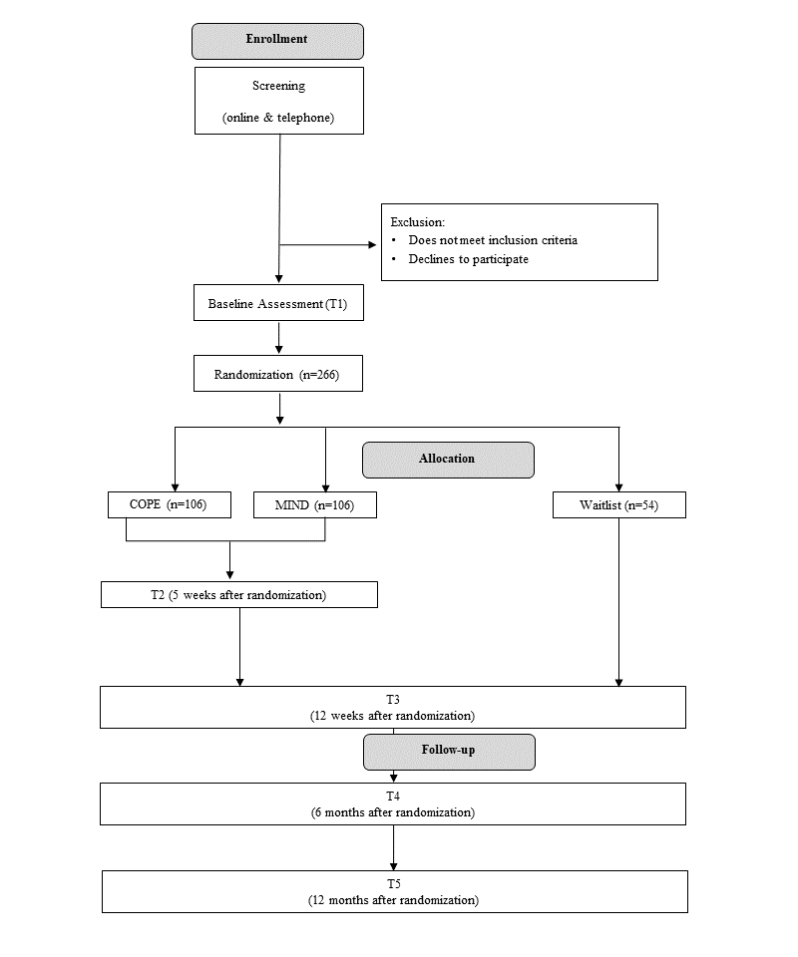
Participant recruitment flowchart.

The study is an RCT of 2 internet-based treatments (ie, I-CBT and I-MBT) versus a waitlist control group. Analyses will be conducted and reported in accordance with the statement by Consolidated Standards of Reporting Trials (CONSORT) [30]. Recommendations for the reporting of psychological and eHealth interventions will be considered [31,32]. This 3-arm superiority trial with 2:2:1 allocation ratio will demonstrate whether the benefit from I-CBT or I-MBT is superior to natural remission of low desire symptoms. Outcomes will be assessed at baseline (T1), at midtreatment (T2, 5 weeks after randomization, only in active conditions), at posttreatment (T3, 12 weeks after randomization), and at 6 months (T4) and 12 months (T5) after randomization. Two computerized reaction-time paradigms will be administered at T1 and T3. Participants will receive up to 3 reminders for each assessment (after 7, 14, and 21 days). Qualitative telephone interviews with active participants will be conducted at T3. For an overview of participant flow, see Figure 1.

### Participants

#### Inclusion and Exclusion Criteria

Inclusion criteria are being 18 years or older; completion of the informed consent form; female gender; being able to read, write, and speak German; and meeting ICD-11 criteria for HSDD. Exclusion criteria are being pregnant, ongoing treatment for any sexual dysfunction or plans to enter such treatment, symptoms of a physical condition that might interfere with study participation (eg, cancer, multiple sclerosis), current substance use disorder, current or lifetime schizophrenia spectrum or other psychotic disorders, and significant relationship discord or violence. As sexual dysfunctions show high rates of comorbidity with other mental health issues such as anxiety or affective disorders [7,33], women experiencing mild to moderate symptoms of other mental disorders are not excluded automatically. Rather, symptoms of a mental disorder (eg, eating disorders, obsessive-compulsive disorder, posttraumatic stress disorder, major depression, bipolar disorder) that might interfere with participation (ie, might make it too burdensome for individuals to participate in data assessments) are assessed during screening. Women whose low desire may be fully attributable to pain during sexual intercourse are also not included. Inclusion and exclusion criteria will be assessed in a 2-step process consisting of an online screening followed by an in-depth telephone interview with a clinical psychologist.

#### Procedure

In order to recruit a diverse sample of women of different ages, sexual orientations, and ethnicities, a variety of recruitment strategies will be employed. Women can learn about the study by visiting a designated study website and associated pages on social networks, articles in local and nationwide media publications (eg, magazines, newspapers), online discussion boards, and flyers at specialized counseling agencies/sites, general practitioners, and gynecologists.

Recruitment and study enrollment will follow a stepwise procedure. Potential participants will be invited to complete an online questionnaire assessing inclusion and exclusion criteria. Individuals not meeting the study criteria (eg, male gender, no sexual concerns, or minors) will be informed and, if needed, be referred to other resources (eg, their physician, counseling services). For all other individuals, an in-depth telephone interview with a clinical psychologist will be scheduled to evaluate eligibility. If an individual is eligible, she will receive an invitation to an internet-based questionnaire. Women who complete this baseline assessment and who provide informed consent electronically will be enrolled.

#### Randomization

Enrolled participants will be randomly assigned to 1 of the 3 conditions. To maximize the power of the study to detect differences between active and control conditions, quotas are applied to allocate 40% of participants to each of the active treatments and 20% to the waitlist. A stratification procedure will be applied to balance relationship status (partner vs no partner) and age (younger than 30 years vs 31 years and older). These variables were selected based on their known effect on sexual desire [34-36]. An internet-based randomization tool will be used by a trained research assistant not involved in the recruitment, screening, or treatment of participants [37]. The clinical psychologist conducting the diagnostic interviews will not be involved in the randomization process but will be informed about the assigned condition after its completion. Block randomization with varying block-sizes will be applied. Participants will be informed about their assigned condition. Participants in the active conditions will gain immediate access to the respective program. Participants assigned to waitlist will receive access to a program of their choice (ie, I-CBT or I-MBT) 6 months after randomization.

### Power Analysis

Sample size was determined based on an a priori power analysis and practical considerations. Psychological therapies for sexual dysfunctions in women yield large effects for symptom improvement compared to waitlist controls (*d*≈0.9) [15,29]. To use a conservative estimate, a medium effect of *d*=0.6 was expected for both active conditions compared to the waitlist (unpaired *t* test two-sided, α=.025, 1–β=80%, allocation rate 2/1). In addition, a postsample of 81 women for each of the 2 active conditions would yield statistical power of 60%, 71%, and 81% to detect differences of *d*=0.35, 0.40, or 0.45, respectively. Although this study is not sufficiently powered to detect smaller differences in efficacy between I-CBT and I-MBT, it will still provide first estimates that may be useful for future studies. As internet-based interventions commonly suffer from relatively high attrition rates, a 30% loss at postassessment analysis is expected. Based on these considerations, a total of 266 women will be enrolled in the study.

### Interventions

#### Structure and Content of Both Interventions

The study introduces 2 internet-based, guided, self-help interventions for low sexual desire in women. To allow for a clear communication with participants, the I-CBT and I-MBT programs are entitled COPE and MIND, respectively. COPE and MIND consist of 8 modules each; women are encouraged to complete 1 module per week. Four weeks after completion of Module 8, participants will gain access to an additional booster module. For a more detailed overview of the modules, see Table 1. Modules are designed to be completed by most participants within 45-60 minutes. Participants are free to take additional time for completion, if needed. As an optional feature, participants are invited to keep an internet-based daily diary of their mood, level of sexual desire, and the time spent with at-home exercises each day. This diary can be accessed via a corresponding smartphone app. Both COPE and MIND have been developed based on existing face-to-face group treatments for low sexual desire (Brotto, Paterson, Basson, Driscoll, and Grabovac, unpublished data, 2015) as well as an internet-based intervention for women with genito-pelvic pain/penetration disorder [38]. If participants fail to log in to the platform for 7 consecutive days, they will receive up to 4 reminders (after 7, 14, 21, and approximately 30 days) by their eCoach and the study coordinator.

In each module, participants receive new information and are introduced to new at-home exercises to be completed between modules. Self-exploration (Modules 2-4) and sensate focus exercises (Modules 6-8) as well as information on the prevalence of sexual dysfunctions, genital anatomy, and sexual response (Modules 1-3) are included in both COPE and MIND. In Module 2, participants will be introduced to the circular sexual response model as proposed by Basson [39] and in each of the following modules, different parts of the cycle will be presented. This model proposes that women with low desire tend to enter sexual situations from a state of neutrality and, if context and stimulation are adequate, may develop sexual arousal and responsive sexual desire during the course of a (pleasing) sexual encounter. (For more information, see Basson [40]). Participants are given the opportunity to work with their sexual response cycle, to reflect on their own situation (eg, to identify contexts they find sexually arousing, to explore reasons for sexual activity), and to develop strategies to improve their sexual experiences.

**Table 1:** Content of the modules.

Module	Content		
	MIND	COPE	Sex therapy (for both interventions)
1	Introduction to mindfulness	Introduction to cognitive behavioral model and body image	Psychoeducation, information about psychological and physiological aspects of sexual response
2	Being present in the moment: Formal and informal mindfulness	Sexual myths	Self-exploration, body exposure
3	Bodily sensations, sitting meditation	Thought protocol, cognitive distortions	Self-exploration, body exposure
4	Mindfulness towards thoughts	Situational analysis	Self-exploration with touch
5	Being present during sexual activity	Vertical arrow technique	Self-exploration with touch
6	Letting go	Cognitive restructuring	Sensate focus exercises
7	Detached awareness	Cognitive restructuring	Sensate focus exercises
8	Addressing difficulties	Working with cognitive methods after the intervention	Summary of Modules 1-7

#### COPE Program (I-CBT)

The focus of COPE are cognitive behavioral techniques that are commonly used in the treatment of mental health problems such as depression and anxiety. In Module 3, participants will learn about automatic thoughts and cognitive distortions [41] and will receive information about the relationship between cognitions, emotions, and behavior. Participants will be asked to identify their automatic thoughts about sexuality, sexual desire, and sexual relationships and will be encouraged to pinpoint underlying thought distortions. In Modules 3 and 4, participants will learn about situational analysis [42,43], and will be guided to apply this method to a personal situation relevant to their low desire (eg, declining a partner’s sexual initiative). Situational analysis can be used to improve understanding of the bidirectional relationship between inner experiences (ie, thoughts and feelings) and overt behavior (ie, what someone does or says) in interpersonal situations. It can be a means to identify problematic thought and behavior patterns and encourages individuals to think about strategies for more positive outcomes in future situations. In Module 5, participants will be introduced to the concept of maladaptive cognitive schemas. They will learn about underlying processes and the way in which schemas develop over the lifespan. They will be asked to identify their own negative concepts related to low sexual desire using the downward arrow technique [41]. Building on content of previous modules, challenging schemas and developing alternative thoughts will part of Modules 6 and 7.

#### MIND Program (I-MBT)

MIND focusses on mindfulness as a therapeutic concept and covers several formal and informal mindfulness-based exercises (eg, body scan, sitting meditation, or mindful eating) adapted from established mindfulness treatments for chronic depression, stress [44], and sexual dysfunctions [45]. Every module starts with a short mindfulness exercises, a so-called Check-In, that invites participants to mindfully focus on different targets such as the breath, negative thoughts, or bodily sensations. In Module 1, participants will learn about the concept of mindfulness in general and will learn about formal mindfulness exercises. In Module 2, they will be introduced to informal mindfulness exercises and breathing meditation. In Module 3, participants will learn detachment from negative thoughts and bodily sensations, as well as acceptance of negative thoughts and emotions. With the progressing mindfulness practice, participants will be encouraged to focus on detachment from all bodily, cognitive, and emotional experiences (Modules 4-6). In Modules 7 and 8, participants will be enabled to consolidate the newly acquired skills into an everyday mindfulness practice.

#### Treatment Platform

The technology platform used to deliver the interventions is provided by Minddistrict. This company is responsible for the provision and maintenance of the platform. Its content management system is used to upload the interventions, add new participants, and eCoaches. Access to the platform is provided by a combination of email and personalized password. The platform conforms to all required quality standards and operates according to the ISO 27000 and NEN 7510 norms. All data are securely stored on ISO 27000-certified servers and transmitted using HTTPS with SSL certificates (AES-256 and SHA-1, 2048-bit RSA).

#### Module Format

Each module is built as a series of webpages with each page featuring text-based information, illustrations, audio, or video clips. Three case vignettes are revisited throughout the interventions to provide examples of how different women may benefit from particular aspects of the treatments and how difficulties with certain exercises can be overcome. Additional content (eg, summary of scientific findings) can be accessed by participants who are interested to learn more about a certain topic. Each module is introduced by a summary of the previous content and an overview of the content to follow. A list of all at-home exercises and a short overview of the completed module are provided at the end of all modules. All worksheets and audio files included in the interventions can be accessed over the platform itself. In addition, participants can access study materials via a link to an external online hosting service.

#### Coaching

eCoaches are female graduate psychology students, specifically trained for the study and supervised by the study coordinator (MM). eCoaches are informed as soon as a participant that is allocated to their account has completed a module. Within the next 48 hours, they provide an individual feedback based on the participants’ answers. To increase comparability between eCoaches, they follow guidelines previously outlined for the coaching process. These guidelines were developed based on the existing literature [46,47] and aim to enable eCoaches to establish a therapeutic alliance and a productive therapeutic working environment. Feedback should include empathetic reassurance, unconditional positive regard, guidance on exercises, and encouragement of openness to new experiences. All eCoaches will be supervised by the study coordinator (MM), a clinical psychologist.

### Measures

#### Primary Outcome

The primary outcome is sexual desire, as measured by the Sexual Interest and Desire Inventory-Female (SIDI-F) [48]. The SIDI-F is a 13-item assessment tool validated for use with clinical populations. The item domains assess frequency and intensity of sexual desire and arousal over the past month [49]. An adapted self-report version, called SIDI-F-SR, will be used to assess sexual desire at all assessment points. This self-report scale has been used in studies with women with distressing low sexual desire [17,50] and was found to have good internal consistency. The SIDI-F-SR has shown high agreement with the SIDI-F (ICC 0.86) in a subsample (n=170) of this study. When corrections for the restriction of range were applied, internal consistency of the SIDI-F-SR was 0.91. Test–retest reliability over a period of 14 weeks was good (*r*=0.74) [51].

#### Secondary Outcome

The Female Sexual Distress Scale Revised (FSDS-R) [52] is a validated 13-item measure used extensively in treatment outcome studies [53], with 1 item specifically assessing sex-related distress due to low sexual desire. The FSDS-R covers the frequency of negative cognitions or emotions women experience with regard to their sexual life overall, sexual problems, or sexual relationships. Items are rated on a 5-point Likert-scale (never to always), resulting in a total score ranging from 0-52. Higher scores indicate higher levels of distress. In previous studies, the FSDS-R has displayed good discriminant validity, high test–retest reliability, and high internal consistency [52].

#### Other Measures

Other measures will comprise sociodemographic variables (eg, age, education, relationship status, and duration), symptom checklists, and a standardized clinical interview for diagnoses according to DSM-5 [54,55]. For an overview of measures, see Table 2. Relationship satisfaction will be assessed with the Relationship Assessment Scale [56]. Sexual communication will be assessed with the Dyadic Sexual Communication Scale [57,58] and mindfulness, body image, and body connection will be assessed by the Mindful Attention and Awareness Scale [59], Body Image Self-Consciousness Scale [60], and the Scale of Body Connection [61], respectively. Possible adverse effects of the interventions will be assessed with the Inventory for the Assessment of Negative Effects of Psychotherapy [62]. Experiences with traumatic events or childhood abuse will be assessed with a subsection of the Childhood Trauma Questionnaire [63-65]. Levels of depression and general anxiety will be measured by the Patient Health Questionnaire 9 [66] and the Generalized Anxiety Disorder 7 [67], respectively. Sexual excitation and sexual inhibition will be assessed with a short version of the Sexual Inhibition/Sexual Excitation Scales [68,69]. Compassionate attitude toward one self will be measured by the Self-Compassion Scale [70]. The level of involvement with thoughts about sexuality will be assessed by the Rumination subscale of the Rumination-Reflection Questionnaire [71,72]. Reasons for drop-out, help-seeking behavior, and reasons for nonutilization of health care for sexual problems will be measured with self-developed items. The relationship between eCoach and participant will be measured with the Helping Alliance Questionnaire [73], whereas overall satisfaction with the program will be measured with an adapted version of the Client Satisfaction Questionnaire [74].

**Table 2:** Overview of measures.

Measure		Timepoint					
		T0	T1	T2	T3	T4	T5
Sexual Interest and Desire Inventory-Female (self-report version: SIDI-F-SR)		—^a^	X	X	X	X	X
Female Sexual Distress Scale Revised		—	X	X	X	X	X
**Other measures (in alphabetical order)**							
	Body Image Self-Consciousness Scale	—	X	—	X	X	X
	Childhood Trauma Questionnaire (Subscales for Sexual, Physical, and Emotional Abuse)	X	X	—	—	—	—
	Client Satisfaction Questionnaire adapted to internet-based interventions (CSQ-I)	—	—	X	X	—	X
	Diagnosis checklist for sexual dysfunctions (ICD-10^b^ and DSM-5^c^)	X	—	—	—	—	—
	Dyadic Sexual Communication Scale	—	X	—	X	X	X
	Generalized Anxiety Disorder 7	—	X	—	X	X	X
	Inventory for the Assessment of Negative Effects of Psychotherapy	—	—	—	X	X	X
	Mindful Attention and Awareness Scale	—	X	—	X	X	X
	Patient Health Questionnaire 9	—	X	—	X	X	X
	Potential reasons for dropout	—	—	—	X	X	X
	Relationship Assessment Scale	—	X	—	X	X	X
	Rumination-Reflection Questionnaire	—	X	—	X	X	X
	Scale of Body Connection	—	X	—	X	X	X
	Self-Compassion Scale	—	X	—	X	X	X
	Sexual Inhibition/Sexual Excitation Scales (short version)	—	X	—	X	X	X
	Sociodemographic questionnaire	X	X	—	X	X	X
	Utilization of additional help	—	—	—	X	X	X

^a^Dashes indicate that the measure will not be assessed.

^b^ICD-10: tenth edition of the International Classification of Diseases.

^c^DSM: fifth edition of the Diagnostic and Statistical Manual of Mental Disorders.

#### Qualitative Interviews

To gain insights into idiosyncratic factors influencing adherence and acceptance of COPE and MIND, structured telephone interviews will be conducted. Twelve weeks after randomization, participants in active conditions who have accessed at least four modules will be invited to take part in the interview until 25 interviews per active condition are completed. The interview questions cover topics such as experiences with the program, perceived changes and improvements, and their view on helpfulness of the coaching (see Multimedia Appendix 1 for a list of interview questions). Interviews will be conducted by trained graduate psychology students not involved in the eCoaching of the respective participant or the study coordinator (MM).

#### Experimental Tasks

To assess the impact of the interventions on sexuality-related interpretations and associations, participants will be asked to complete 2 reaction-time paradigms at T1 and T3. To complete these paradigms, they will be asked to download a small computer program (ie, Inquisit 5 Web App) to their computer. Instructions on how to install the program will be provided in an email and on the download website. Sexuality-related associations will be assessed with a STIAT, an experimental task prompting participants to categorize stimuli into different categories as quickly as possible [75,76]. Reaction times are used as a behavioral reference toward the presented stimuli material. The underlying assumption of the task is that strongly associated stimuli are processed more quickly compared to weakly associated stimuli, as reflected in participants’ categorization speed (eg, faster reaction times when sexual pictures are paired with the category "I don't want" as compared to "I want"). Sexuality-related interpretations will be assessed with an SST that requires participants to sort 6 words presented on a computer screen (eg, “desire I sexual often feel seldom”) into a grammatically correct sentence, using only 5 words [77,78]. Depending on the omitted word, the resulting sentence can have a positive (eg, “I often feel sexual desire”) or negative valence (eg, “I seldom feel sexual desire”). A study using a convenience sample of women with mixed levels of sexual desire suggested that lower levels of desire are associated with more negative sentences in the SST [79].

### Statistical Analyses

Analyses will be conducted and reported according to the CONSORT guidelines [30]. An intention-to-treat approach will be employed to analyze data. Missing data will be handled using multiple imputations [80]. In addition, study completer analyses including only participants who completed data assessments and intervention completer analyses including only participants who completed all treatment modules will be conducted. Differences in the primary and secondary outcomes between each of the active conditions (ie, I-CBT and I-MBT) and the waitlist control group will be examined with repeated measures ANOVA, and standardized effect sizes (Cohen *d*) and their respective confidence intervals will be calculated for comparison. Predictors and moderators of change will be analyzed via structural equation modeling on an exploratory basis. For statistical analyses, the significance level will be set at *P*<.05, 2-sided, or *P*<.025, 2-sided, in cases where 2 parallel analyses for each of the active conditions are conducted.

## Results

Recruitment has begun in January 2019 and is expected to be completed in August 2021. Results will be published in 2022. We anticipate improvements in our primary (ie, sexual desire as measured with the SIDI-F-SR) and secondary outcomes (ie, sexual distress as measured with the FSDS-R) from pretreatment to posttreatment in the I-CBT and I-MBT conditions compared to the waitlist. These improvements are expected to be sustained at 6- and 12-month follow-up. No significant difference in outcomes between active conditions is expected. However, potential differences in efficacy between active conditions will be explored, and the trial will be sufficiently powered to identify medium to large differences between I-CBT and I-MBT.

## Discussion

### Protocol Overview

Low sexual desire negatively affects millions of women and their partners. Psychological interventions are effective to improve sexual desire and sexual satisfaction [15] but only a minority of women have access to qualified care providers (ie, sexual therapists). Evaluating the efficacy of psychological internet-based interventions is key to solve this problem and to provide help for women in need. Both cognitive behavioral and mindfulness-based treatments are commonly used to treat sexual dysfunctions [15]. To assess whether these therapeutic approaches are feasible and effective when delivered via the internet and to explore for which groups of patients one or both of the treatments are most beneficial are the goals of this trial.

### Strategies for Mitigating Challenges

With regard to potential challenges, nonadherence to treatment and attrition are common problems that affect studies of psychological treatments in general, and of internet-based interventions in particular. To address this issue, we will include extensive adherence reminders via email and telephone as well as guidance by eCoaches, which has been linked to higher treatment adherence in internet-based interventions [81]. eCoaches are instructed to closely monitor potential adverse effects (eg, deterioration of symptoms) in participants. If a woman experiences any adverse effects, eCoaches may offer guidance and help in the form of additional text messages via the treatment platform. In more serious cases (ie, a participant experiencing severe distress), the study coordinator (MM) may contact the participant via phone to provide further support. In Module 8, participants can also access information on further treatment options (eg, face-to-face therapy). Moreover, we will assess potential side effects of the treatment with the Inventory for the Assessment of Negative Effects of Psychotherapy at T3 and T4 [62]. In order to ensure high quality care and to provide help in difficult situations (eg, in dealing with dissatisfied participants), eCoaches will receive continuous support by the study coordinator (MM).

### Study Contribution

This study will close an important gap in the literature and will show whether cognitive behavioral and mindfulness-based internet interventions are effective in improving low desire in women with HSDD. While uncontrolled and small-scale studies point in this direction, this study will add to the literature by employing a larger sample of women and including longer follow-up intervals. Thus, our study will provide first evidence of whether treatment effects can be sustained over several months. If the results for I-CBT and/or I-MBT are promising, this study should contribute to an improvement of the care situation for women with low sexual desire. Comprehensive data assessments pretreatment, peritreatment, and posttreatment will allow us to investigate moderators and mediators of change and provide important information on *how* I-CBT and I-MBT are working.

### Limitations

This study is sufficiently powered to allow for a comparison of I-CBT and I-MBT to a waitlist control group for our primary outcome measure (ie, the SIDI-F-SR). Thus, it will not allow us to identify smaller differences in efficacy between the 2 active conditions or subtle changes in other variables. Although efforts have been made to create study content that should be appropriate and helpful for a wide variety of participants (eg, optional content, case vignettes with women of different ages, partnership status, and sexual orientations), not all women may find the interventions suited for their needs. All treatment modules include audiovisual content (eg, infographics, videos, audio files) to supplement text-based information; still, women with reading difficulties or non-German speakers might find study participation difficult. The same problem applies to the data assessments which mostly rely on internet-based self-report questionnaires. Some participants may need up to 50 minutes to complete data assessments which require a high level of commitment and concentration and place an additional burden on participants.

### Conclusion

This study will be the first comprehensive RCT to investigate the efficacy of I-CBT and I-MBT for the treatment of HSDD in women. It will provide insights into new applications of evidence-based treatments, helping to disseminate new options to women, who would not have sought out other treatments due lack of access to sexual therapists or fear of stigmatization. The study findings may also shed light on potential mechanisms of change and help to improve clinical decision making for women with HSDD.

## Appendix

Multimedia Appendix 1 Follow-up interview.

Multimedia Appendix 2 Peer review report.

## Abbreviations

CBT: cognitive behavioral therapy
CONSORT: Consolidated Standards of Reporting Trials
DSM: fifth edition of the Diagnostic and Statistical Manual of Mental Disorders
FSDS-R: Female Sexual Distress Scale Revised
HSDD: hypoactive sexual desire dysfunction (formerly hypoactive sexual desire disorder)
I-CBT: internet-based CBT
ICD-10: tenth edition of the International Classification of Diseases
I-MBT: internet-based MBT
MBT: mindfulness-based therapy
RCT: randomized controlled trial
SIDI-F: Sexual Interest and Desire Inventory-Female
SST: Scrambled Sentences Task
STIAT: Single-Target Implicit Association Test
