# Supplementary material for: Psychological Treatment of Low Sexual Desire in Women: Protocol for a Randomized, Waitlist-Controlled Trial of Internet-Based Cognitive Behavioral and Mindfulness-Based Treatments
Source: JMIR Res Protoc. 2020 Sep 29;9(9):e20326. doi: 10.2196/20326 (PMC7556380; doi:10.2196/20326)
Supplement: Multimedia Appendix 1 [file resprot_v9i9e20326_app1.docx]

**Follow-up interview**

| **Nr.** | **Frage** | **Question** |
| --- | --- | --- |
| 1 | An welcher Variante des Programms haben Sie teilgenommen? | Which version of the program did you participate in? |
| 2 | Was hat Ihnen persönlich die Teilnahme am Programm gebracht? | What have you personally gained from participating in the intervention? |
| 3 | Welche Veränderungen haben Sie selbst erlebt?  [Wenn Veränderungen angegeben]   1. Wie geht es Ihnen mit diesen Veränderungen? 2. Worauf führen Sie diese Veränderungen zurück? | What changes have you experienced?  [If changes are reported]   1. How do you feel about these changes? 2. In your opinion, what has caused these changes? |
| 4 | Welche Bestandteile des Programms haben Sie als hilfreich empfunden? | Which parts of the intervention did you find helpful? |
| 5 | Jeweils für die verschiedenen Hauptbestandteile der Übungen (KVT, MBT, Sexualtherapie):   1. Welche dieser Übungen fanden Sie besonders hilfreich? 2. Was daran fanden Sie besonders hilfreich? | For each kind of exercise (CBT, MBT and sex therapy):   1. Which of these exercises did you find particularly helpful? 2. What about these exercises did you find particularly helpful? |
| 6 | 1. Gibt es etwas, das Sie sich im Programm zusätzlich gewünscht hätten? 2. Wenn Sie an Ihre Erfahrungen mit dem Programm denken, worauf hätten Sie am ehesten verzichten können? 3. Haben Sie konkrete Verbesserungsvorschläge für Teile des Programms? | 1. Is there anything you would have liked to see added to the program? 2. Thinking about your experiences with the program: Which elements of the intervention would have been dispensible for you? 3. Do you have specific suggestions for how to improve the program? |
| 7 | 1. Was hat Ihnen die Begleitung durch Ihre eCoachin gebracht? 2. Gibt es etwas, das Sie sich von der eCoachin zusätzlich gewünscht hätten? 3. Haben Sie konkrete Verbesserungsvorschläge für das eCoaching? 4. Welche Rückmeldungen bzw. Anmerkungen der eCoachin waren für Sie besonders hilfreich? | 1. What has the support of your eCoach done for you? 2. Is there anything else you would have liked from the eCoach? 3. Do you have specific suggestions for how to improve the eCoaching? 4. Which kinds of feedback or comments from the eCoach were particularly helpful to you? |
| 8 | Sind bei Ihnen während der Teilnahme am Programm Schwierigkeiten aufgetreten?  [Wenn ja]   1. Wie sind Sie damit umgegangen? | Have you encountered difficulties while participating in the program?  [If endorsed]   1. How did you cope with these? |
| 9 | 1. Welche Strategien haben Sie verwendet, regelmäßig am Ball zu bleiben?   [Wenn Nachfrage nötig]   1. Wie haben Sie das geschafft, sich zu motivieren? 2. Welche Hindernisse gab es für Sie, regelmäßig die Lektionen zu bearbeiten? 3. Welche Unterstützung, Hilfen oder andere Dinge hätten es Ihnen leichter gemacht regelmäßig teilzunehmen? | 1. Which strategies have you employed to take part in the intervention regularly?   [If further inquiry needed]   1. How did you manage to motivate yourself? 2. What were the obstacles for you to work through the lessons regularly? 3. Which support, aids or other things would have made it easier for you to participate regularly? |
| 10 | 1. Warum haben Sie sich für die Teilnahme an der Studie entschieden? 2. Können Sie sich zurückerinnern und beschreiben, welche Erwartungen Sie an das Programm hatten?   [Wenn Erwartungen genannt]   1. Wurden diese Erwartungen erfüllt? | 1. Why did you participate in the study? 2. Do you remember any of your expectations back from before you started the program?   [If expectations are mentioned]   1. Were these expectations met? |
| 11 | 1. Was sind für Sie im Allgemeinen die Vorteile oder Stärken von internetbasierter Behandlung sexueller Schwierigkeiten? 2. Und welche Nachteile oder Schwächen sehen Sie von internetbasierter Behandlung sexueller Schwierigkeiten? | 1. In general, what do you think are the advantages or strengths of Internet-based treatments of sexual difficulties? 2. And what are the disadvantages or weaknesses of Internet-based treatments of sexual difficulties? |
| 12 | Sind Sie aktuell in einer Partnerschaft?  [Wenn ja]   1. Wie haben Sie Ihren Partner bzw. Ihre Partnerin in das Programm mit einbezogen? 2. Wie hat Ihr Partner bzw. Ihre Partnerin auf die Teilnahme reagiert? 3. Haben Sie konkrete Vorschläge, wie man den Einbezug eines Partners bzw. einer Partnerin in einem solchen Onlineprogramm noch verbessern könnte?   [Wenn nein]   1. Vielleicht werden Sie ja in Zukunft eine neue Partnerschaft eingehen wollen. Was denken Sie, hat die Teilnahme am Programm Ihnen in Bezug auf eine zukünftige Partnerschaft gebracht? | Are you currently in a relationship?  [If endorsed]   1. How did you involve your partner in the program? 2. How did your partner react to your participation? 3. Do you have specific suggestions on how to improve the involvement of a partner in such an online program?   [If no]   1. Let’s assume that you want to enter a new relationship in the future. What do you think your participation in the program has brought you in terms of a future relationship? |
| 13 | Gibt es noch etwas, was Sie gerne ansprechen oder ergänzen würden, wonach ich Sie bisher nicht gefragt habe? | Is there anything else you would like to add? |
