# Supplementary material for: Psychological Treatment of Low Sexual Desire in Women: Protocol for a Randomized, Waitlist-Controlled Trial of Internet-Based Cognitive Behavioral and Mindfulness-Based Treatments
Source: JMIR Res Protoc. 2020 Sep 29;9(9):e20326. doi: 10.2196/20326 (PMC7556380; doi:10.2196/20326)
Supplement: Multimedia Appendix 2 [file resprot_v9i9e20326_app2.pdf]

RUHR UNIVERSITÄT BOCHUM RUB

Deutsche Forschungsgemeinschaft
Geistes- und Sozialwissenschaften
Kennedy Allee 40
53175 Bonn

Fakultät für Psychologie
Forschungs- und Behandlungszentrum für psychische Gesundheit
Dr. Julia Velten
Massenbergstr. 9-13, 44787 Bochum
☏ 0049 (0) 234 - 32 27939
💻 julia.velten@rub.de

Bochum, 07.11.17

Einreichung des überarbeiteten Antrages VE 1083/1-1

Sehr geehrte Frau Dr. Kollei,

wie mit Frau Klein besprochen, möchte ich die Gelegenheit nutzen, eine überarbeitete Version des o. g. Antrages zur Begutachtung bei der DFG einzureichen.

Ich möchte mich zunächst für das sehr hilfreiche und konstruktive Feedback der GutachterInnen und des Fachgremiums bedanken. Gemeinsam mit Herrn Prof. Margraf und Frau Prof. Brotto habe ich die Rückmeldungen diskutiert und ein neues Studienkonzept entwickelt. Durch die Überarbeitung des Designs und Modifikation der geplanten Behandlungsarme war es möglich, die Kritikpunkte aufzugreifen und die klinische Relevanz und wissenschaftliche Qualität des Antrages zu erhöhen.

Auf den folgenden Seiten nehme ich Punkt für Punkt Stellung zu den Anmerkungen der GutachterInnen bzw. des Fachgremiums.

Ich freue mich auf eine erneute Begutachtung meines Antrages.

Mit freundlichen kollegialen Grüßen

Julia Velten

Julia Velten

1. Gutachten (auf Englisch)

Reviewer 1 describes the clinical significance of our project and underlines the potential of Internet-based interventions to reduce self-stigmatization in women with sexual dysfunctions. She/He acknowledges the connections of our research group to leading international experts, but asks about the individual contributions of these experts.

- The applicant has revised the section on page 20 to describe the role of these experts in more detail. All contributors have agreed to support the applicant during all stages of this study. Prof. Brotto, for example, has reviewed earlier drafts of this proposal and is involved in adapting her offline group-interventions for the use in an Internet-based treatment. She is an expert in mindfulness-therapy and will guide the applicant on how to adapt mindfulness exercises to the online-environment.
- The applicant was also able to win Prof. van Lankveld as an additional advisor for this project. Prof. van Lankveld is an expert in online-treatments of sexual dysfunctions and will advise the applicant on all issues concerning the online-intervention.
- Prof. Margraf is the head of the Department of Clinical Psychology and Psychotherapy at the RUB and an expert in clinical psychology with longstanding experience in the facilitation of clinical trials. He will also be involved throughout the whole project.

Reviewer 1 acknowledges that the power analysis concerning the waitlist control-group is well done, but has some concerns about the assumed medium-sized effect between the two treatment arms.

- The applicant has discussed this issue with her colleagues and agrees that it may not be realistic to expect that the group-format improves our treatment outcome to such a large extent.
- In the light of this and other comments by Reviewer 1 and the review board, we have decided to drop the proposed comparison between individual and group-interventions and decided to focus on a comparison of different treatment-approaches. Our revised proposal compares two Internet-based individual treatments, namely cognitive-behavioral sexual therapy (COPE-program) and mindfulness-based sexual therapy (MIND-program) for women with Hypoactive Sexual Desire Disorder (HSDD) with a waitlist control. In our opinion, this comparison is of greater clinical significance and is based on significant research findings that emphasize the relevance of both treatments for sexual dysfunctions in general.
- The new power analysis is based on a medium effect (d = 0.6) for both active treatments compared to the waitlist. Although we do not expect a significant difference between the two active conditions, our study would be sufficiently powered to identify possible medium to small group differences between MIND and COPE arms.
- Please see page 6ff of the application for an overview of our revised study design and methods.

Reviewer 1 also criticizes that no specific-cutoff on a measure of sexual desire is mentioned as inclusion criterion

- A cutoff of < 34 in our primary endpoint, the Sexual Interest and Desire Inventory – Female (SIDI-F) will be used as inclusion criterion as recommended by Clayton et al., 2010, J Women Health. Please see Page 10 for more information.

Reviewer 1 also inquired about our missing-data strategy.

- We are planning on conducting a comprehensive missing-data analysis and base our strategy on the pattern of missing data as data replacement strategies vary between data missing at random (MAR) and data not missing at random (MNAR).
- Together with our statistician Ms. Zhang, we will apply appropriate strategies based, for example, on the recommendations by *Graham, J. W. (2009). Missing Data Analysis: Making It Work in the Real World. Annual Review of Psychology, 549–576.*

Reviewer 1 has questioned the feasibility of the study and has asked about a breakdown of numbers of earlier stages of the study (e.g., how many women will visit the website, how many women will not sign consent).

- A recent study in the UK showed that 7% of women feel distressed by recent low sexual desire (Burri et al., 2011, J Sex Med). Another study showed that less than half of sexually dysfunctional women seek help for this problem. However, if only 1% of the approximately 30 Million adult women currently living in Germany, seek help for low sexual desire, this would lead to 300.000 potential participants.
- The following two studies/projects underline the feasibility of our recruitment numbers:
    - The applicant recently started a sexual counselling service at RUB. During the last month, Google advertisement were presented to 1,200 individuals in the Ruhr-Metropolitan area who were searching for sexual therapy or counselling for a sexual problem online. About 15 % of these individuals (*n* = 86) clicked on our ad and visited our website. More than 11 % (*n* = 10) scheduled an appointment at our clinic. During clinical interviews, one or more sexual dysfunctions were confirmed in nine of these cases. These numbers show that online advertisements for sexual counselling services are effective and suggest that the proposed recruitment numbers are feasible.
    - According to Anna-Carlotta Zarski, a researcher who is facilitating an online-study for women with Genito-Pelvic Pain/Penetration Disorder, a sexual pain condition, 356 women contacted her to inquire about the study in a 17 months period. 138 of these women (39%) were enrolled in the study. However, Ms. Zarski did not use extensive Google advertisements and was offering treatment for a sexual pain condition that is much less common than low sexual desire in women.
- At this point, we are not able to provide more detailed information about the potential number of people who visit our website and are not interested in participating. This is also difficult to assess as we are not saving IP-addresses and thus, cannot differentiate whether one individual is visiting the website ten times or ten individuals just once.

Reviewer 1 also raised ethical concerns about participants that are not capable of filling out questionnaires online or do not have access to the Internet.

- Unfortunately, we are unable to deliver our treatments offline or to provide an alternative intervention for participants without Internet-access. In addition, minimal experience with the use of Internet-websites (e.g., how to send an email, how to navigate websites) is needed to participate.
- However, we will design our interventions in a very user-friendly way. We will use brief sentences and will visualize content using pictures, figures, or videos. We will also create our programs in a way that makes it easy for participants to navigate through the content. Our online-counsellors will also support participants who express difficulties in using the website.

2. Gutachten

Das zweite Gutachten beschreibt die Stärken unseres Antrages und die klinische Relevanz des Projektes. Zusätzlich wird die Konsultation eines Experten für Online-Interventionen empfohlen.

- Wir haben Kontakt zu Herrn Prof. van Lankveld aufgenommen, der uns in Bezug auf die Gestaltung der Programm-Seiten sowie der Durchführung der Online-Studie beraten wird. Zudem steht die Antragstellerin in engem Austausch mit Frau Zarski von der Universität Nürnberg-Erlangen, die derzeit eine Online-Studie zu sexuellen Schmerzen durchführt. Mithilfe dieser Experten wird es möglich sein, potentielle Schwierigkeiten in Bezug auf die Durchführung zu überwinden bzw. die geplanten Interventionen auf bestmögliche Weise umzusetzen. Die genaue Zusammenarbeit mit den Experten wird auf Seite 20 im Antrag dargestellt.

Fachkollegium

Das Fachkollegium weist darauf hin, dass beim Erstantrag keine Differenzierung zwischen Frauen mit Verlust an sexuellem Verlangen und Frauen mit Erregungsstörungen vorgenommen wird.

- Die Frage danach, ob es sinnvoller ist, Störungen des sexuellen Verlangens und Erregungsstörungen bei Frauen durch ein (DSM-5) oder zwei Störungsbilder (ICD-10 und 11) zu beschreiben, wird aktuell in Fachkreisen intensiv diskutiert. Nach Diskussion mit meinen KollegInnen, stimmen wir jedoch zu, dass eine Vermischung dieser beiden Symptombereiche die Aussagekraft der Studie reduziert.
- Um diesem Kritikpunkt Rechnung zu tragen, fokussieren wir uns im revidierten Studiendesign auf Frauen, deren Hauptbeschwerden sich auf den Verlust oder den Mangel an sexuellem Verlangen (ICD-10 F52.0) beziehen. Epidemiologische Studien (z. B. West et al., 2008, Arch Intern Med) sowie Übersichtsarbeiten (z. B. Brotto, 2017, Front Neuroendocrinol) beschreiben übereinstimmend, dass hypoaktives sexuelles Verlangen ein sehr belastendes und schwierig zu behandelndes Störungsbild bei Frauen darstellt.
- Das Vorliegen von komorbiden sexuellen Störungen (z. B. Orgasmus- oder Lubrikationsstörungen) ist kein Ausschlusskriterium und wird im Rahmen der Diagnostik mit erhoben. Während die geplanten Interventionen speziell auf das Thema hypoaktives sexuelles Verlangen ausgerichtet werden, erscheint wahrscheinlich, dass auch komorbide sexuelle Störungsbereiche durch unsere Interventionen verbessert werden können.

Das Fachkollegium weist darauf hin, dass keine deutlichen Unterschiede in der Wirksamkeit von Einzel- und Gruppentherapie erwartet werden können.

- Wie bereits geschildert, stimmen wir zu, dass dieser Vergleich mit dem im Erstantrag beschriebenen Studiendesign nicht hinreichend umgesetzt werden kann. Gemeinsam mit Prof. Margraf und Prof. Brotto hat die Antragstellerin daher entschieden, den Fokus des geplanten Projektes zu verändern und zwei aktive Einzelbehandlungen (Kognitiv-behaviorale Sexualtherapie vs. Achtsamkeitsbasierte Sexualtherapie) miteinander zu vergleichen. Die Umsetzbarkeit sowie die klinische Relevanz kann unserer Auffassung nach durch die Veränderung des Studienfokus deutlich gesteigert werden.
- Während Prof. Margraf als Experte für die Kognitive Verhaltenstherapie gelten kann, ist mit Prof. Brotto eine Expertin für die achtsamkeitsbasierte Sexualtherapie als Kollaborationspartnerin mit an Bord. Beide Therapieverfahren sind in Offline-Therapien für fehlendes sexuelles Verlangen für wirksam befunden, jedoch (a) noch nicht in einem RCT vergleichend untersucht und (b) noch nicht als Online-Therapie evaluiert worden.

- Durch unser verändertes Studiendesign wird es zudem möglich sein, mehr über die Wirkmechanismen sowie die differenzielle Wirksamkeit beider Interventionen bei unterschiedlichen Patientinnen-Gruppen herauszufinden. Die Forschung dazu steckt noch in den Kinderschuhen, so dass wir bislang wenig darüber wissen, welche Therapieverfahren z. B. bei Frauen, die depressive Symptome zeigen wirkungsvoller sind.

Das Fachkollegium weist darauf hin, dass die Erhebungsmethoden genauer hätten beschrieben werden können.

- Im überarbeiteten Antrag werden die verwendeten Erhebungsinstrumente genauer beschrieben. Angaben zur psychometrischen Qualität der wesentlichen Verfahren wurden ergänzt (siehe Seite 8f).

Zum Schluss greift das Fachkollegium noch den Punkt der klinischen Relevanz auf und stellt in Frage, inwiefern (a) durch Anzeigen eine klinisch beeinträchtigte Stichprobe erreicht werden kann und (b) wie sich die Aufwandsentschädigung auswirkt.

- Zu (a): Durch die Rückmeldungen von FachkollegInnen sowie eigene Erfahrungen mit der neu eingerichteten Sexualsprechstunde an der Hochschulambulanz der Ruhr-Universität Bochum sind wir sehr zuversichtlich, dass durch unsere Rekrutierungsstrategie eine klinisch beeinträchtige Population zu erreicht werden kann (siehe Seite 2 dieses Schreibens). Da sich viele Betroffene über das Internet informieren, können viele klinisch-beeinträchtigte Probandinnen über Google-Anzeigen gewonnen werden. Um die Repräsentativität unserer Stichprobe für die Gesamtheit aller Betroffenen zu erhöhen, werden wir darüber hinaus noch klassische Rekrutierungswege z. B. über Arztpraxen wählen. Eine genaue Beschreibung unserer Rekrutierungsstrategie findet sich auf Seite 10 des Antrages.
- Die Durchführung der Online-Intervention an sich ist für die Teilnehmerinnen kostenfrei und wird auch nicht vergütet. Um jedoch eine umfassende Evaluation des Forschungsprojektes sicherzustellen, ist ein nicht unerheblicher Aufwand für zusätzliche Datenerhebungen von den Teilnehmerinnen zu leisten. Um eine sorgfältige Mitwirkung an den Datenerhebungen gerade auch zu den Follow-Up Zeiträumen einige Monate nach der Behandlung sicherzustellen, erscheint uns eine Aufwandsentschädigung das probate Mittel.
- Wir haben uns bei der Höhe und Staffelung der Aufwandsentschädigungen an den Richtlinien internationaler Ethikkommissionen orientiert (z. B. http://www.irb.vt.edu/pages/compensation.htm). Mit einem Stundenlohn von 10 Euro für die Datenerhebungen wurde die Vergütung so festgelegt, dass es eine unzulässige Beeinflussung der Probandinnen unwahrscheinlich erscheint. Die Vergütung ist zudem nicht von der Beendigung der Studie abhängig, sondern wird nach jeder abgeschlossenen Datenerhebung ausgezahlt.
